# Exercise and Over-Exercise

**Published:** 1899-02-04

**Authors:** 


### Exercise and Over-Exercise.

In an address delivered by Dr. Lauder Brunton before the York Medical Society, and printed in the *Quarterly Medical Journal*, the benefits of exercise and the dangers of over-exercise are well described. It is pointed out that in every way exercise tends to increased nutrition and to greater growth. *Punch*, who with marvellous impartiality, has for so long portrayed, week by week, the varying aspects of society, is an oracle who may fitly be consulted in regard to the effects of that muscular exercise which for the last twenty years has played so large a part in the education of girls. "It is very interesting and instructive," says Dr. Brunton, "to compare the sketches in *Punch* of girls playing croquet, by Leech, and a game of lawn tennis, by Du Maurier. In Leech's sketches we see that the girls are smaller, the exertion they make is slight, and their movements are not only stiff, but they exercise only a few muscles; while Du Maurier's girls are tall, strong, lithe, and graceful, and, in striking the ball, put every muscle in their body into active exercise. Now, in lawn tennis or polo there is more general movement of the whole body than in croquet, cricket, or golf, and it is just possible that the increased growth in recent years of girls, as compared with their brothers, may be due to more general exercise of the muscles in lawn tennis by them, as compared with the more restricted action in playing cricket."

If, then, we accept the broad fact that exercise is good, and that its effect is to increase muscular strength and bodily development, we have to ask, Why does still further exercise cease to be good, and what do we mean by over-exercise? First, we must draw a broad distinction between the effect of constantly-maintained exertion which is required in certain trades, and the alternating contraction and relaxation of all the muscles of the body, which is the characteristic of games and athletics of all kinds. That constantly-maintained effort or monotonous repetition of the same movement tends to exhaustion rather than nutrition goes without saying; but taking ordinary so-called healthy exercise, by what is its limit set? The limit appears to be set by three factors: (1) The capacity of the digestive organs to keep up the quality of the blood; (2) the capacity of the excretory organs to get rid of the waste products which result from muscular action; and (3) the power of the heart to drive a constant stream of blood through every corner of the organism.

Interference with digestion is a by no means uncommon effect of excessive exercise, and so far as training is concerned, it is one of the most destructive. The blood cannot flow in full stream to every part at once. As Dr. Lauder Brunton says, "Everyone knows that while moderate exercise tends to produce appetite, a long and exhausting exertion tends to destroy the appetite, and even to produce actual sickness, as one finds in mountain climbing." People differ greatly in this respect but in some—great ponderous men as they may seem—the digestion is so easily upset by muscular exercise that, although they may be giants for a momentary exertion, anything like sustained effort disturbs digestion, and cuts at the very root of their nutrition.

In many cases, however, the limit to exercise lies in diminished excretion. Unless the excretory organs are thoroughly efficient the tissues become crowded with products which cannot be got rid of, the senses become dimmed, and effort becomes a mere automatism, in consequence of a self-poisoning by the products of muscular waste.

So far we have dealt with what may be fitly termed the automatic checks to over exercise. Interference with digestion so lowers nutrition, while accumulation of waste products so poisons the system that in either case further exertion becomes impossible—the very will to make it passes away. But it is different in regard to the heart. The heart, although strained, may yet be driven on to its own destruction. Every muscular effort not only demands from the heart an increased flow of blood, but also drives an increased quantity towards it. So long as the heart can pass this forward all is well, but when it fails not merely is the circulation of the blood rendered imperfect, but serious damage is done to the heart itself. If when the heart was over driven it merely struck, the enfeebled circulation would soon put a stop to further effort. The willing heart, however, taking at each beat a wider sweep, and driving into the vessels a larger quantity of blood, so meets the call that the athlete can struggle on, perhaps to win his race. But the strained heart suffers, the stretched muscle does not quite come back, the dilated cavity does not quite close at each contraction, and permanent mischief is set up. Thus it is that exercise driven to the limit imposed by the heart is over exercise in the most serious sense of the word. If it is the heart that stops it, the chances are that it has already gone too far.

